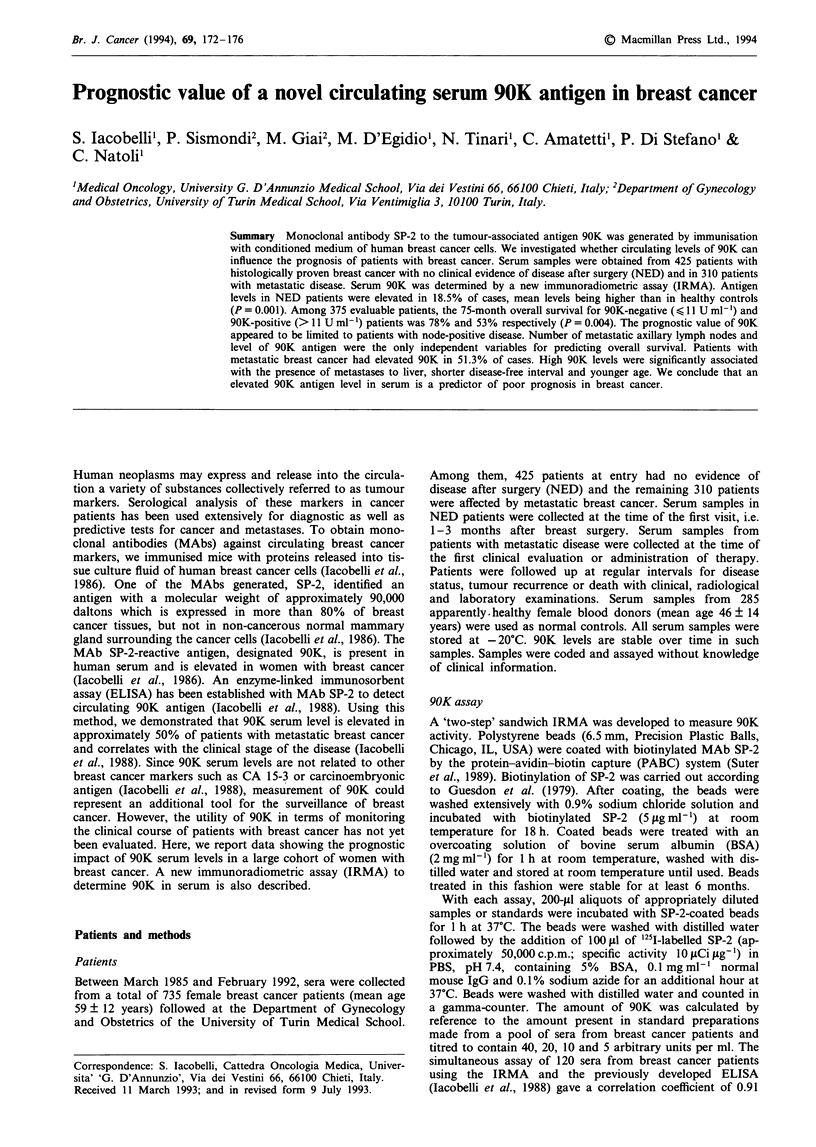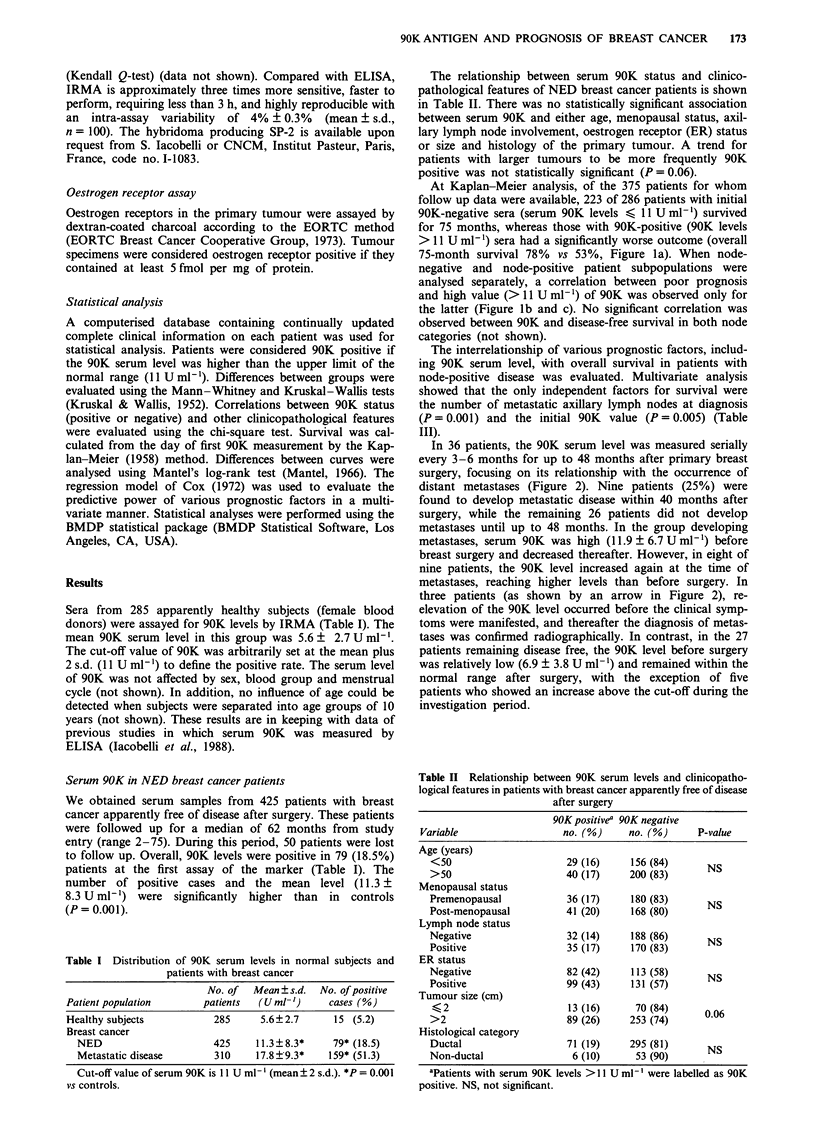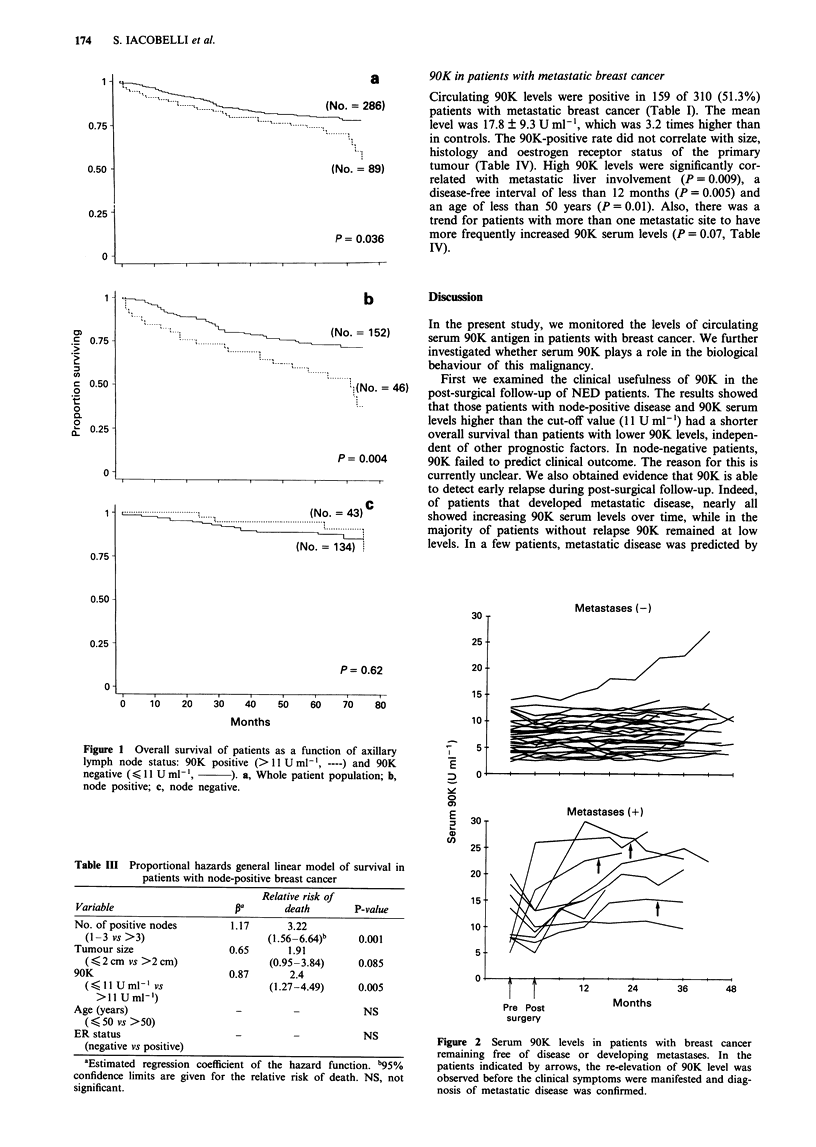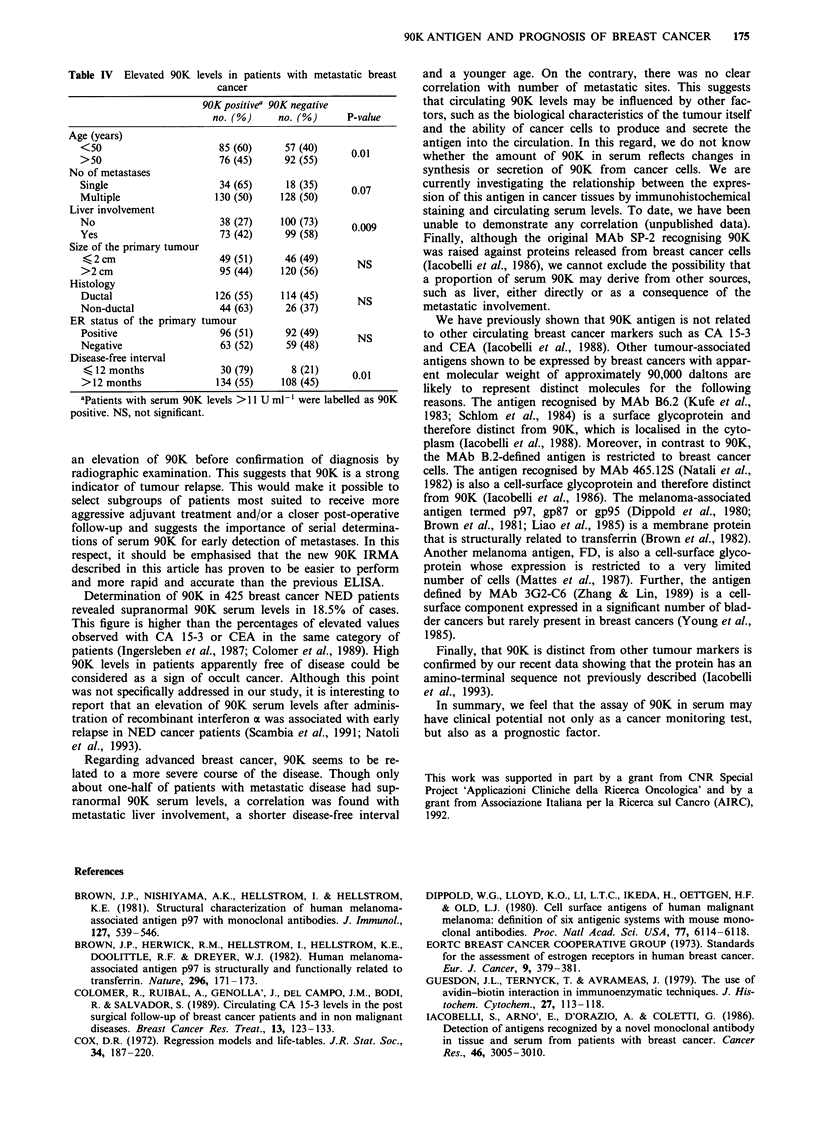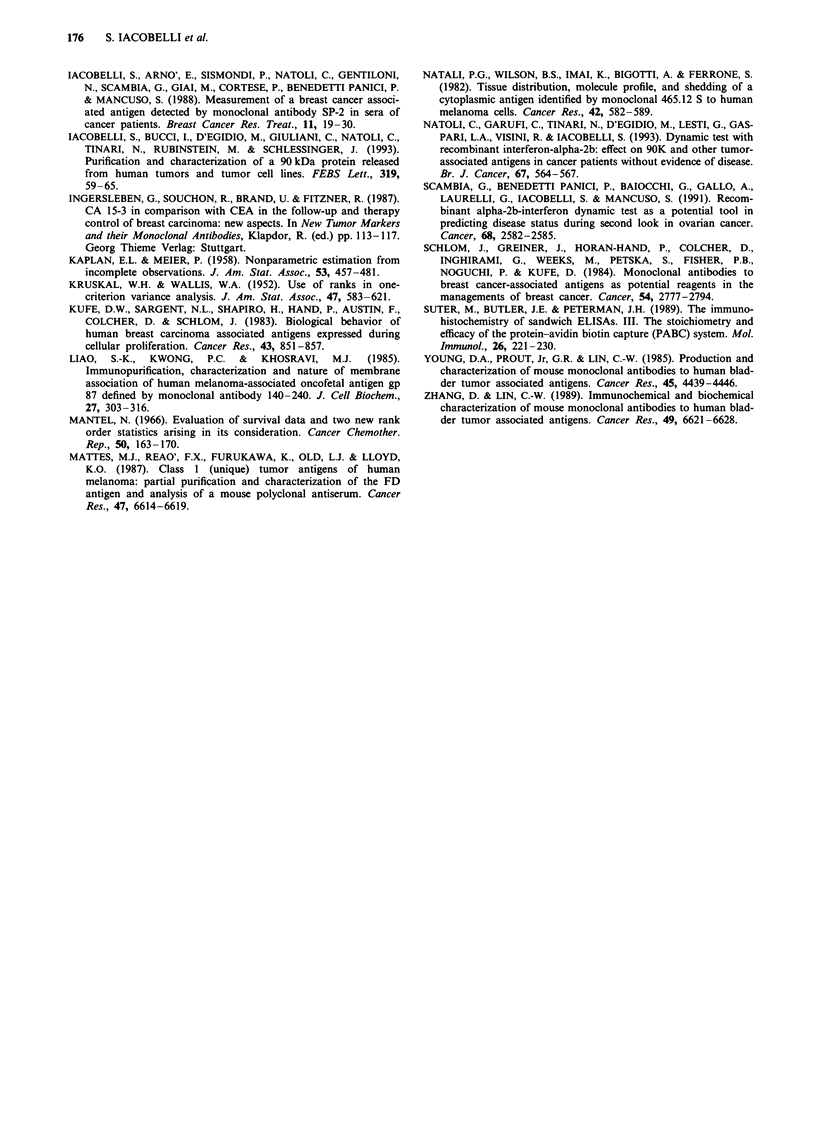# Prognostic value of a novel circulating serum 90K antigen in breast cancer.

**DOI:** 10.1038/bjc.1994.29

**Published:** 1994-01

**Authors:** S. Iacobelli, P. Sismondi, M. Giai, M. D'Egidio, N. Tinari, C. Amatetti, P. Di Stefano, C. Natoli

**Affiliations:** University G. D'Annunzio Medical School, Chieti, Italy.

## Abstract

Monoclonal antibody SP-2 to the tumour-associated antigen 90K was generated by immunisation with conditioned medium of human breast cancer cells. We investigated whether circulating levels of 90K can influence the prognosis of patients with breast cancer. Serum samples were obtained from 425 patients with histologically proven breast cancer with no clinical evidence of disease after surgery (NED) and in 310 patients with metastatic disease. Serum 90K was determined by a new immunoradiometric assay (IRMA). Antigen levels in NED patients were elevated in 18.5% of cases, mean levels being higher than in healthy controls (P = 0.001). Among 375 evaluable patients, the 75-month overall survival for 90K-negative (< or = 11 U ml-1) and 90K-positive (> 11 U ml-1) patients was 78% and 53% respectively (P = 0.004). The prognostic value of 90K appeared to be limited to patients with node-positive disease. Number of metastatic axillary lymph nodes and level of 90K antigen were the only independent variables for predicting overall survival. Patients with metastatic breast cancer had elevated 90K in 51.3% of cases. High 90K levels were significantly associated with the presence of metastases to liver, shorter disease-free interval and younger age. We conclude that an elevated 90K antigen level in serum is a predictor of poor prognosis in breast cancer.


Br. J. Cancer (1994), 69, 172-176 © Macmillan Press Ltd., 1994

### Prognostic value of a novel circulating serum 90K antigen in breast cancer

S. Iacobelli[1], P. Sismondi[2], M. Giai[2], M. D'Egidio[1], N. Tinari[1], C. Amatetti[1], P. Di Stefano[1] & C. Natoli[1]

[1]*Medical Oncology, University G. D'Annunzio Medical School, Via dei Vestini 66, 66100 Chieti, Italy;* [2]*Department of Gynecology and Obstetrics, University of Turin Medical School, Via Ventimiglia 3, 10100 Turin, Italy.*

**Summary** Monoclonal antibody SP-2 to the tumour-associated antigen 90K was generated by immunisation with conditioned medium of human breast cancer cells. We investigated whether circulating levels of 90K can influence the prognosis of patients with breast cancer. Serum samples were obtained from 425 patients with histologically proven breast cancer with no clinical evidence of disease after surgery (NED) and in 310 patients with metastatic disease. Serum 90K was determined by a new immunoradiometric assay (IRMA). Antigen levels in NED patients were elevated in 18.5% of cases, mean levels being higher than in healthy controls ($P = 0.001$). Among 375 evaluable patients, the 75-month overall survival for 90K-negative ($\leqslant 11$ U $ml^{-1}$) and 90K-positive ($> 11$ U $ml^{-1}$) patients was 78% and 53% respectively ($P = 0.004$). The prognostic value of 90K appeared to be limited to patients with node-positive disease. Number of metastatic axillary lymph nodes and level of 90K antigen were the only independent variables for predicting overall survival. Patients with metastatic breast cancer had elevated 90K in 51.3% of cases. High 90K levels were significantly associated with the presence of metastases to liver, shorter disease-free interval and younger age. We conclude that an elevated 90K antigen level in serum is a predictor of poor prognosis in breast cancer.

Human neoplasms may express and release into the circulation a variety of substances collectively referred to as tumour markers. Serological analysis of these markers in cancer patients has been used extensively for diagnostic as well as predictive tests for cancer and metastases. To obtain monoclonal antibodies (MAbs) against circulating breast cancer markers, we immunised mice with proteins released into tissue culture fluid of human breast cancer cells (Iacobelli *et al.*, 1986). One of the MAbs generated, SP-2, identified an antigen with a molecular weight of approximately 90,000 daltons which is expressed in more than 80% of breast cancer tissues, but not in non-cancerous normal mammary gland surrounding the cancer cells (Iacobelli *et al.*, 1986). The MAb SP-2-reactive antigen, designated 90K, is present in human serum and is elevated in women with breast cancer (Iacobelli *et al.*, 1986). An enzyme-linked immunosorbent assay (ELISA) has been established with MAb SP-2 to detect circulating 90K antigen (Iacobelli *et al.*, 1988). Using this method, we demonstrated that 90K serum level is elevated in approximately 50% of patients with metastatic breast cancer and correlates with the clinical stage of the disease (Iacobelli *et al.*, 1988). Since 90K serum levels are not related to other breast cancer markers such as CA 15-3 or carcinoembryonic antigen (Iacobelli *et al.*, 1988), measurement of 90K could represent an additional tool for the surveillance of breast cancer. However, the utility of 90K in terms of monitoring the clinical course of patients with breast cancer has not yet been evaluated. Here, we report data showing the prognostic impact of 90K serum levels in a large cohort of women with breast cancer. A new immunoradiometric assay (IRMA) to determine 90K in serum is also described.

### Patients and methods

### *Patients*

Between March 1985 and February 1992, sera were collected from a total of 735 female breast cancer patients (mean age 59 ± 12 years) followed at the Department of Gynecology and Obstetrics of the University of Turin Medical School. Among them, 425 patients at entry had no evidence of disease after surgery (NED) and the remaining 310 patients were affected by metastatic breast cancer. Serum samples in NED patients were collected at the time of the first visit, i.e. 1-3 months after breast surgery. Serum samples from patients with metastatic disease were collected at the time of the first clinical evaluation or administration of therapy. Patients were followed up at regular intervals for disease status, tumour recurrence or death with clinical, radiological and laboratory examinations. Serum samples from 285 apparently healthy female blood donors (mean age 46 ± 14 years) were used as normal controls. All serum samples were stored at −20°C. 90K levels are stable over time in such samples. Samples were coded and assayed without knowledge of clinical information.

### *90K assay*

A 'two-step' sandwich IRMA was developed to measure 90K activity. Polystyrene beads (6.5 mm, Precision Plastic Balls, Chicago, IL, USA) were coated with biotinylated MAb SP-2 by the protein-avidin-biotin capture (PABC) system (Suter *et al.*, 1989). Biotinylation of SP-2 was carried out according to Guesdon *et al.* (1979). After coating, the beads were washed extensively with 0.9% sodium chloride solution and incubated with biotinylated SP-2 (5 $\mu g$ $ml^{-1}$) at room temperature for 18 h. Coated beads were treated with an overcoating solution of bovine serum albumin (BSA) (2 mg $ml^{-1}$) for 1 h at room temperature, washed with distilled water and stored at room temperature until used. Beads treated in this fashion were stable for at least 6 months.

With each assay, 200-$\mu l$ aliquots of appropriately diluted samples or standards were incubated with SP-2-coated beads for 1 h at 37°C. The beads were washed with distilled water followed by the addition of 100 $\mu l$ of $^{125}I$-labelled SP-2 (approximately 50,000 c.p.m.; specific activity 10 $\mu Ci$ $\mu g^{-1}$) in PBS, pH 7.4, containing 5% BSA, 0.1 mg $ml^{-1}$ normal mouse IgG and 0.1% sodium azide for an additional hour at 37°C. Beads were washed with distilled water and counted in a gamma-counter. The amount of 90K was calculated by reference to the amount present in standard preparations made from a pool of sera from breast cancer patients and titred to contain 40, 20, 10 and 5 arbitrary units per ml. The simultaneous assay of 120 sera from breast cancer patients using the IRMA and the previously developed ELISA (Iacobelli *et al.*, 1988) gave a correlation coefficient of 0.91

Correspondence: S. Iacobelli, Cattedra Oncologia Medica, Universita' 'G. D'Annunzio', Via dei Vestini 66, 66100 Chieti, Italy.
Received 11 March 1993; and in revised form 9 July 1993.

(Kendall $Q$-test) (data not shown). Compared with ELISA, IRMA is approximately three times more sensitive, faster to perform, requiring less than 3 h, and highly reproducible with an intra-assay variability of 4% ± 0.3% (mean ± s.d., $n = 100$). The hybridoma producing SP-2 is available upon request from S. Iacobelli or CNCM, Institut Pasteur, Paris, France, code no. I-1083.

*Oestrogen receptor assay*

Oestrogen receptors in the primary tumour were assayed by dextran-coated charcoal according to the EORTC method (EORTC Breast Cancer Cooperative Group, 1973). Tumour specimens were considered oestrogen receptor positive if they contained at least 5 fmol per mg of protein.

*Statistical analysis*

A computerised database containing continually updated complete clinical information on each patient was used for statistical analysis. Patients were considered 90K positive if the 90K serum level was higher than the upper limit of the normal range (11 U ml$^{-1}$). Differences between groups were evaluated using the Mann–Whitney and Kruskal–Wallis tests (Kruskal & Wallis, 1952). Correlations between 90K status (positive or negative) and other clinicopathological features were evaluated using the chi-square test. Survival was calculated from the day of first 90K measurement by the Kaplan–Meier (1958) method. Differences between curves were analysed using Mantel's log-rank test (Mantel, 1966). The regression model of Cox (1972) was used to evaluate the predictive power of various prognostic factors in a multivariate manner. Statistical analyses were performed using the BMDP statistical package (BMDP Statistical Software, Los Angeles, CA, USA).

### Results

Sera from 285 apparently healthy subjects (female blood donors) were assayed for 90K levels by IRMA (Table I). The mean 90K serum level in this group was 5.6 ± 2.7 U ml$^{-1}$. The cut-off value of 90K was arbitrarily set at the mean plus 2 s.d. (11 U ml$^{-1}$) to define the positive rate. The serum level of 90K was not affected by sex, blood group and menstrual cycle (not shown). In addition, no influence of age could be detected when subjects were separated into age groups of 10 years (not shown). These results are in keeping with data of previous studies in which serum 90K was measured by ELISA (Iacobelli *et al.*, 1988).

*Serum 90K in NED breast cancer patients*

We obtained serum samples from 425 patients with breast cancer apparently free of disease after surgery. These patients were followed up for a median of 62 months from study entry (range 2–75). During this period, 50 patients were lost to follow up. Overall, 90K levels were positive in 79 (18.5%) patients at the first assay of the marker (Table I). The number of positive cases and the mean level (11.3 ± 8.3 U ml$^{-1}$) were significantly higher than in controls ($P = 0.001$).

**Table I** Distribution of 90K serum levels in normal subjects and patients with breast cancer

| *Patient population* | *No. of patients* | *Mean ± s.d. (U ml$^{-1}$)* | *No. of positive cases (%)* |
|---|---|---|---|
| Healthy subjects | 285 | 5.6±2.7 | 15 (5.2) |
| Breast cancer | | | |
| NED | 425 | 11.3±8.3* | 79* (18.5) |
| Metastatic disease | 310 | 17.8±9.3* | 159* (51.3) |

Cut-off value of serum 90K is 11 U ml$^{-1}$ (mean ± 2 s.d.). *$P = 0.001$ *vs* controls.

The relationship between serum 90K status and clinicopathological features of NED breast cancer patients is shown in Table II. There was no statistically significant association between serum 90K and either age, menopausal status, axillary lymph node involvement, oestrogen receptor (ER) status or size and histology of the primary tumour. A trend for patients with larger tumours to be more frequently 90K positive was not statistically significant ($P = 0.06$).

At Kaplan–Meier analysis, of the 375 patients for whom follow up data were available, 223 of 286 patients with initial 90K-negative sera (serum 90K levels $\leqslant$ 11 U ml$^{-1}$) survived for 75 months, whereas those with 90K-positive (90K levels > 11 U ml$^{-1}$) sera had a significantly worse outcome (overall 75-month survival 78% *vs* 53%, Figure 1a). When node-negative and node-positive patient subpopulations were analysed separately, a correlation between poor prognosis and high value (> 11 U ml$^{-1}$) of 90K was observed only for the latter (Figure 1b and c). No significant correlation was observed between 90K and disease-free survival in both node categories (not shown).

The interrelationship of various prognostic factors, including 90K serum level, with overall survival in patients with node-positive disease was evaluated. Multivariate analysis showed that the only independent factors for survival were the number of metastatic axillary lymph nodes at diagnosis ($P = 0.001$) and the initial 90K value ($P = 0.005$) (Table III).

In 36 patients, the 90K serum level was measured serially every 3–6 months for up to 48 months after primary breast surgery, focusing on its relationship with the occurrence of distant metastases (Figure 2). Nine patients (25%) were found to develop metastatic disease within 40 months after surgery, while the remaining 26 patients did not develop metastases until up to 48 months. In the group developing metastases, serum 90K was high (11.9 ± 6.7 U ml$^{-1}$) before breast surgery and decreased thereafter. However, in eight of nine patients, the 90K level increased again at the time of metastases, reaching higher levels than before surgery. In three patients (as shown by an arrow in Figure 2), re-elevation of the 90K level occurred before the clinical symptoms were manifested, and thereafter the diagnosis of metastases was confirmed radiographically. In contrast, in the 27 patients remaining disease free, the 90K level before surgery was relatively low (6.9 ± 3.8 U ml$^{-1}$) and remained within the normal range after surgery, with the exception of five patients who showed an increase above the cut-off during the investigation period.

**Table II** Relationship between 90K serum levels and clinicopathological features in patients with breast cancer apparently free of disease after surgery

| *Variable* | *90K positive[a] no. (%)* | *90K negative no. (%)* | P-*value* |
|---|---|---|---|
| Age (years) | | | |
| <50 | 29 (16) | 156 (84) | NS |
| >50 | 40 (17) | 200 (83) | |
| Menopausal status | | | |
| Premenopausal | 36 (17) | 180 (83) | NS |
| Post-menopausal | 41 (20) | 168 (80) | |
| Lymph node status | | | |
| Negative | 32 (14) | 188 (86) | NS |
| Positive | 35 (17) | 170 (83) | |
| ER status | | | |
| Negative | 82 (42) | 113 (58) | NS |
| Positive | 99 (43) | 131 (57) | |
| Tumour size (cm) | | | |
| $\leqslant$2 | 13 (16) | 70 (84) | 0.06 |
| >2 | 89 (26) | 253 (74) | |
| Histological category | | | |
| Ductal | 71 (19) | 295 (81) | NS |
| Non-ductal | 6 (10) | 53 (90) | |

[a]Patients with serum 90K levels >11 U ml$^{-1}$ were labelled as 90K positive. NS, not significant.

**Figure 1** Overall survival of patients as a function of axillary lymph node status: 90K positive (>11 U ml$^{-1}$, ----) and 90K negative (⩽11 U ml$^{-1}$, ———). **a**, Whole patient population; **b**, node positive; **c**, node negative.

**Table III** Proportional hazards general linear model of survival in patients with node-positive breast cancer

| *Variable* | $\beta^a$ | *Relative risk of death* | P-*value* |
|---|---|---|---|
| No. of positive nodes (1–3 *vs* >3) | 1.17 | 3.22 (1.56–6.64)[b] | 0.001 |
| Tumour size (⩽2 cm *vs* >2 cm) | 0.65 | 1.91 (0.95–3.84) | 0.085 |
| 90K (⩽11 U ml$^{-1}$ *vs* >11 U ml$^{-1}$) | 0.87 | 2.4 (1.27–4.49) | 0.005 |
| Age (years) (⩽50 *vs* >50) | – | – | NS |
| ER status (negative *vs* positive) | – | – | NS |

[a]Estimated regression coefficient of the hazard function. [b]95% confidence limits are given for the relative risk of death. NS, not significant.

*90K in patients with metastatic breast cancer*

Circulating 90K levels were positive in 159 of 310 (51.3%) patients with metastatic breast cancer (Table I). The mean level was 17.8 ± 9.3 U ml$^{-1}$, which was 3.2 times higher than in controls. The 90K-positive rate did not correlate with size, histology and oestrogen receptor status of the primary tumour (Table IV). High 90K levels were significantly correlated with metastatic liver involvement ($P = 0.009$), a disease-free interval of less than 12 months ($P = 0.005$) and an age of less than 50 years ($P = 0.01$). Also, there was a trend for patients with more than one metastatic site to have more frequently increased 90K serum levels ($P = 0.07$, Table IV).

### Discussion

In the present study, we monitored the levels of circulating serum 90K antigen in patients with breast cancer. We further investigated whether serum 90K plays a role in the biological behaviour of this malignancy.

First we examined the clinical usefulness of 90K in the post-surgical follow-up of NED patients. The results showed that those patients with node-positive disease and 90K serum levels higher than the cut-off value (11 U ml$^{-1}$) had a shorter overall survival than patients with lower 90K levels, independent of other prognostic factors. In node-negative patients, 90K failed to predict clinical outcome. The reason for this is currently unclear. We also obtained evidence that 90K is able to detect early relapse during post-surgical follow-up. Indeed, of patients that developed metastatic disease, nearly all showed increasing 90K serum levels over time, while in the majority of patients without relapse 90K remained at low levels. In a few patients, metastatic disease was predicted by

**Figure 2** Serum 90K levels in patients with breast cancer remaining free of disease or developing metastases. In the patients indicated by arrows, the re-elevation of 90K level was observed before the clinical symptoms were manifested and diagnosis of metastatic disease was confirmed.

**Table IV** Elevated 90K levels in patients with metastatic breast cancer

| | *90K positive*[a] *no. (%)* | *90K negative no. (%)* | P-*value* |
|---|---|---|---|
| Age (years) | | | |
| <50 | 85 (60) | 57 (40) | 0.01 |
| >50 | 76 (45) | 92 (55) | |
| No of metastases | | | |
| Single | 34 (65) | 18 (35) | 0.07 |
| Multiple | 130 (50) | 128 (50) | |
| Liver involvement | | | |
| No | 38 (27) | 100 (73) | 0.009 |
| Yes | 73 (42) | 99 (58) | |
| Size of the primary tumour | | | |
| ⩽2 cm | 49 (51) | 46 (49) | NS |
| >2 cm | 95 (44) | 120 (56) | |
| Histology | | | |
| Ductal | 126 (55) | 114 (45) | NS |
| Non-ductal | 44 (63) | 26 (37) | |
| ER status of the primary tumour | | | |
| Positive | 96 (51) | 92 (49) | NS |
| Negative | 63 (52) | 59 (48) | |
| Disease-free interval | | | |
| ⩽12 months | 30 (79) | 8 (21) | 0.01 |
| >12 months | 134 (55) | 108 (45) | |

[a]Patients with serum 90K levels >11 U ml$^{-1}$ were labelled as 90K positive. NS, not significant.

an elevation of 90K before confirmation of diagnosis by radiographic examination. This suggests that 90K is a strong indicator of tumour relapse. This would make it possible to select subgroups of patients most suited to receive more aggressive adjuvant treatment and/or a closer post-operative follow-up and suggests the importance of serial determinations of serum 90K for early detection of metastases. In this respect, it should be emphasised that the new 90K IRMA described in this article has proven to be easier to perform and more rapid and accurate than the previous ELISA.

Determination of 90K in 425 breast cancer NED patients revealed supranormal 90K serum levels in 18.5% of cases. This figure is higher than the percentages of elevated values observed with CA 15-3 or CEA in the same category of patients (Ingersleben *et al.*, 1987; Colomer *et al.*, 1989). High 90K levels in patients apparently free of disease could be considered as a sign of occult cancer. Although this point was not specifically addressed in our study, it is interesting to report that an elevation of 90K serum levels after administration of recombinant interferon α was associated with early relapse in NED cancer patients (Scambia *et al.*, 1991; Natoli *et al.*, 1993).

Regarding advanced breast cancer, 90K seems to be related to a more severe course of the disease. Though only about one-half of patients with metastatic disease had supranormal 90K serum levels, a correlation was found with metastatic liver involvement, a shorter disease-free interval and a younger age. On the contrary, there was no clear correlation with number of metastatic sites. This suggests that circulating 90K levels may be influenced by other factors, such as the biological characteristics of the tumour itself and the ability of cancer cells to produce and secrete the antigen into the circulation. In this regard, we do not know whether the amount of 90K in serum reflects changes in synthesis or secretion of 90K from cancer cells. We are currently investigating the relationship between the expression of this antigen in cancer tissues by immunohistochemical staining and circulating serum levels. To date, we have been unable to demonstrate any correlation (unpublished data). Finally, although the original MAb SP-2 recognising 90K was raised against proteins released from breast cancer cells (Iacobelli *et al.*, 1986), we cannot exclude the possibility that a proportion of serum 90K may derive from other sources, such as liver, either directly or as a consequence of the metastatic involvement.

We have previously shown that 90K antigen is not related to other circulating breast cancer markers such as CA 15-3 and CEA (Iacobelli *et al.*, 1988). Other tumour-associated antigens shown to be expressed by breast cancers with apparent molecular weight of approximately 90,000 daltons are likely to represent distinct molecules for the following reasons. The antigen recognised by MAb B6.2 (Kufe *et al.*, 1983; Schlom *et al.*, 1984) is a surface glycoprotein and therefore distinct from 90K, which is localised in the cytoplasm (Iacobelli *et al.*, 1988). Moreover, in contrast to 90K, the MAb B.2-defined antigen is restricted to breast cancer cells. The antigen recognised by MAb 465.12S (Natali *et al.*, 1982) is also a cell-surface glycoprotein and therefore distinct from 90K (Iacobelli *et al.*, 1986). The melanoma-associated antigen termed p97, gp87 or gp95 (Dippold *et al.*, 1980; Brown *et al.*, 1981; Liao *et al.*, 1985) is a membrane protein that is structurally related to transferrin (Brown *et al.*, 1982). Another melanoma antigen, FD, is also a cell-surface glycoprotein whose expression is restricted to a very limited number of cells (Mattes *et al.*, 1987). Further, the antigen defined by MAb 3G2-C6 (Zhang & Lin, 1989) is a cell-surface component expressed in a significant number of bladder cancers but rarely present in breast cancers (Young *et al.*, 1985).

Finally, that 90K is distinct from other tumour markers is confirmed by our recent data showing that the protein has an amino-terminal sequence not previously described (Iacobelli *et al.*, 1993).

In summary, we feel that the assay of 90K in serum may have clinical potential not only as a cancer monitoring test, but also as a prognostic factor.

This work was supported in part by a grant from CNR Special Project 'Applicazioni Cliniche della Ricerca Oncologica' and by a grant from Associazione Italiana per la Ricerca sul Cancro (AIRC), 1992.

### References

BROWN, J.P., NISHIYAMA, A.K., HELLSTROM, I. & HELLSTROM, K.E. (1981). Structural characterization of human melanoma-associated antigen p97 with monoclonal antibodies. *J. Immunol.*, **127**, 539–546.

BROWN, J.P., HERWICK, R.M., HELLSTROM, I., HELLSTROM, K.E., DOOLITTLE, R.F. & DREYER, W.J. (1982). Human melanoma-associated antigen p97 is structurally and functionally related to transferrin. *Nature*, **296**, 171–173.

COLOMER, R., RUIBAL, A., GENOLLA', J., DEL CAMPO, J.M., BODI, R. & SALVADOR, S. (1989). Circulating CA 15-3 levels in the post surgical follow-up of breast cancer patients and in non malignant diseases. *Breast Cancer Res. Treat.*, **13**, 123–133.

COX, D.R. (1972). Regression models and life-tables. *J.R. Stat. Soc.*, **34**, 187–220.

DIPPOLD, W.G., LLOYD, K.O., LI, L.T.C., IKEDA, H., OETTGEN, H.F. & OLD, L.J. (1980). Cell surface antigens of human malignant melanoma: definition of six antigenic systems with mouse monoclonal antibodies. *Proc. Natl Acad. Sci. USA*, **77**, 6114–6118.

EORTC BREAST CANCER COOPERATIVE GROUP (1973). Standards for the assessment of estrogen receptors in human breast cancer. *Eur. J. Cancer*, **9**, 379–381.

GUESDON, J.L., TERNYCK, T. & AVRAMEAS, J. (1979). The use of avidin–biotin interaction in immunoenzymatic techniques. *J. Histochem. Cytochem.*, **27**, 113–118.

IACOBELLI, S., ARNO', E., D'ORAZIO, A. & COLETTI, G. (1986). Detection of antigens recognized by a novel monoclonal antibody in tissue and serum from patients with breast cancer. *Cancer Res.*, **46**, 3005–3010.

IACOBELLI, S., ARNO', E., SISMONDI, P., NATOLI, C., GENTILONI, N., SCAMBIA, G., GIAI, M., CORTESE, P., BENEDETTI PANICI, P. & MANCUSO, S. (1988). Measurement of a breast cancer associated antigen detected by monoclonal antibody SP-2 in sera of cancer patients. *Breast Cancer Res. Treat.*, **11**, 19–30.

IACOBELLI, S., BUCCI, I., D'EGIDIO, M., GIULIANI, C., NATOLI, C., TINARI, N., RUBINSTEIN, M. & SCHLESSINGER, J. (1993). Purification and characterization of a 90 kDa protein released from human tumors and tumor cell lines. *FEBS Lett.*, **319**, 59–65.

INGERSLEBEN, G., SOUCHON, R., BRAND, U. & FITZNER, R. (1987). CA 15-3 in comparison with CEA in the follow-up and therapy control of breast carcinoma: new aspects. In *New Tumor Markers and their Monoclonal Antibodies*, Klapdor, R. (ed.) pp. 113–117. Georg Thieme Verlag: Stuttgart.

KAPLAN, E.L. & MEIER, P. (1958). Nonparametric estimation from incomplete observations. *J. Am. Stat. Assoc.*, **53**, 457–481.

KRUSKAL, W.H. & WALLIS, W.A. (1952). Use of ranks in one-criterion variance analysis. *J. Am. Stat. Assoc.*, **47**, 583–621.

KUFE, D.W., SARGENT, N.L., SHAPIRO, H., HAND, P., AUSTIN, F., COLCHER, D. & SCHLOM, J. (1983). Biological behavior of human breast carcinoma associated antigens expressed during cellular proliferation. *Cancer Res.*, **43**, 851–857.

LIAO, S.-K., KWONG, P.C. & KHOSRAVI, M.J. (1985). Immunopurification, characterization and nature of membrane association of human melanoma-associated oncofetal antigen gp 87 defined by monoclonal antibody 140–240. *J. Cell Biochem.*, **27**, 303–316.

MANTEL, N. (1966). Evaluation of survival data and two new rank order statistics arising in its consideration. *Cancer Chemother. Rep.*, **50**, 163–170.

MATTES, M.J., REAO', F.X., FURUKAWA, K., OLD, L.J. & LLOYD, K.O. (1987). Class 1 (unique) tumor antigens of human melanoma: partial purification and characterization of the FD antigen and analysis of a mouse polyclonal antiserum. *Cancer Res.*, **47**, 6614–6619.

NATALI, P.G., WILSON, B.S., IMAI, K., BIGOTTI, A. & FERRONE, S. (1982). Tissue distribution, molecule profile, and shedding of a cytoplasmic antigen identified by monoclonal 465.12 S to human melanoma cells. *Cancer Res.*, **42**, 582–589.

NATOLI, C., GARUFI, C., TINARI, N., D'EGIDIO, M., LESTI, G., GASPARI, L.A., VISINI, R. & IACOBELLI, S. (1993). Dynamic test with recombinant interferon-alpha-2b: effect on 90K and other tumor-associated antigens in cancer patients without evidence of disease. *Br. J. Cancer*, **67**, 564–567.

SCAMBIA, G., BENEDETTI PANICI, P., BAIOCCHI, G., GALLO, A., LAURELLI, G., IACOBELLI, S. & MANCUSO, S. (1991). Recombinant alpha-2b-interferon dynamic test as a potential tool in predicting disease status during second look in ovarian cancer. *Cancer*, **68**, 2582–2585.

SCHLOM, J., GREINER, J., HORAN-HAND, P., COLCHER, D., INGHIRAMI, G., WEEKS, M., PETSKA, S., FISHER, P.B., NOGUCHI, P. & KUFE, D. (1984). Monoclonal antibodies to breast cancer-associated antigens as potential reagents in the managements of breast cancer. *Cancer*, **54**, 2777–2794.

SUTER, M., BUTLER, J.E. & PETERMAN, J.H. (1989). The immunohistochemistry of sandwich ELISAs. III. The stoichiometry and efficacy of the protein–avidin biotin capture (PABC) system. *Mol. Immunol.*, **26**, 221–230.

YOUNG, D.A., PROUT, Jr, G.R. & LIN, C.-W. (1985). Production and characterization of mouse monoclonal antibodies to human bladder tumor associated antigens. *Cancer Res.*, **45**, 4439–4446.

ZHANG, D. & LIN, C.-W. (1989). Immunochemical and biochemical characterization of mouse monoclonal antibodies to human bladder tumor associated antigens. *Cancer Res.*, **49**, 6621–6628.